# Play the Music, not the Instrument

**DOI:** 10.1007/s40037-014-0116-1

**Published:** 2014-03-20

**Authors:** Jan van Dalen

**Affiliations:** Maastricht University, Maastricht, the Netherlands

It is every Editor’s dream that articles in ‘their’ journal serve a wide population of interested readers and that some papers are so challenging that they are actually followed up by readers’ reactions.

Consequently, the Editors of the current issue of Perspectives on Medical Education are proud of the issue you see on your screen now (or even hold in your hands)!

We have six full papers for you, with contributions from all over the globe. Topics ranging from bedside teaching to feedback are waiting here for you. We have a PhD report, a book review and a letter to the Editor. Moreover, two contributions *comment on* articles in this very issue. Granted: they have been written on invitation, but still…

So the Editors of our journal are happy and proud. Apparently the journal attracts authors from diverse backgrounds and it is beginning to serve this community with a wide variation of interesting stuff.

When looking at the papers in this issue from a helicopter point of view, directly or indirectly almost all have to do with the issues of professional identity, group and individual norms, self-esteem and vulnerability when acquiring this professional identity.

This is coincidental, and it may be indicative of the emerging insight that those in the health professions are a relatively separate community. Apparently we have our own unwritten rules and our initiation rites to make newcomers adapt to the field. During the first years the newcomers report to answer to a ‘calling’. This calling diminishes from the first to the third year, or at least: it is reported less [1, 2]. Do they experience less ‘calling’, or is it less socially acceptable towards the third year to admit that you feel ‘called’?

Juniors in medicine are vulnerable to suffer from mental health problems, burnout or heart rate variability, although no overt relation with stress can be established [3]. The medical core values also seem to be transferred by means of case discussions [4], and in bedside teaching [5, 6]. Once the subjects are allowed into the group this coincides with their greater wellbeing [7].

Seen from these viewpoints, the health care professions bear great resemblance to other important professional groups, such as the military in the army or musicians in an orchestra: initiation rites, insecurity, not a great emphasis on individualism, rules that are not immediately crystal clear to outsiders.

I know too little about the army to illustrate this further, but I do know about music. I was therefore extra thrilled to see the piece that Watling [8] contributed to this issue of our journal. In an introduction to his PhD, a comparison was made between the training of doctors and the training of musicians. Differences between the training of doctors and musicians were addressed in his dissertation. I was intrigued to read about these differences. However, there are many similarities in the two professions. Not only in the process (theory integrated with skills, collaboration) but also in the demands (high stakes, social adjustment, competence or even perfection). I would strongly want to encourage Watling and co-authors to focus a next paper on the *similarities* between these two types of training. If we could combine the best of these worlds, maybe this would lead to health professionals who would be excellent craftsmen, handling their instruments with great expertise, flexibility and agility, while these instrumentalists would also be great listeners and interpreters and collaborate to achieve harmony.

I’d say: a lot to be gained in medicine!
